# Therapeutic efficacy of shenmai injection as an adjuvant treatment in dilated cardiomyopathy: A protocol for systematic review

**DOI:** 10.1097/MD.0000000000019158

**Published:** 2020-02-21

**Authors:** Kai Gao, Yan-Ping Song, Anna Song, Hao Chen, Lin-Tao Zhao, Hai-Wang Zhang

**Affiliations:** a Pharmacy College, Shaanxi University of Chinese Medicine, Xianyang; b Shaanxi Academy of Traditional Chinese Medicine, Xi’an, Shaanxi, China; c Michigan State University, East Lansing, Michigan.

**Keywords:** adjuvant treatment, dilated cardiomyopathy, meta-analysis, protocol, shenmai injection, systematic review

## Abstract

**Background:**

Shenmai injection (SMI) is a Traditional Chinese Medicine patent prescription consisting of extractions from ophiopogonis radix and ginseng radix rubra. Clinical studies showed that SMI combined with conventional medicine treatment (CMT) can enhance the therapeutic efficacy for dilated cardiomyopathy (DCM). However, there is still a lack of comprehensive and systematic evidence, which urgently requires us to verify its therapeutic efficacy. Hence, we provide a protocol for systematic review and meta-analysis.

**Methods:**

The systematic search on the MEDLINE/PubMed, China National Knowledge Infrastructure (CNKI), Wanfang database, VIP database, the Cochrane Library, Embase and Chinese Biomedical Database (CBM) in Chinese and English language with dates ranging from the earliest record to August 8, 2019. Next, the quality of each trial was assessed according to the criteria of the Cochrane Handbook for Systematic Reviews of Interventions. Then, the outcome data were recorded and pooled by RevMan 5.3 software.

**Results:**

The systematic review and meta-analysis aims to review and pool current clinical outcomes of SMI for the adjuvant treatment of DCM.

**Conclusion:**

This study will provide a high-quality evidence of SMI for the adjuvant treatment on DCM patients.

**PROSPERO Registration Number:**

CRD42019146369.

## 1 Introduction

Dilated cardiomyopathy (DCM), the most common form of primary or secondary cardiac muscle disease, which is characterized by ventricular dilation and ventricular dysfunction.^[1]^ DCM accounts for about 60% of all cardiomyopathies, and is generally considered to be the ultimate common response of the cardiac muscle to many genetic and environmental acquired injuries.^[2]^ It is the most common cardiomyopathy among children. According to a large epidemiological study, the incidence of infants (<1 year old) and older children were 4.40 and 0.34 cases per 100,000 people per year, respectively.^[3]^ In adults, the prevalence is 1 in 2500 individuals, with an incidence of 7 per 100,000 per year.^[4]^ However, due to a lack of understanding of the etiology and pathophysiology of DCM, it persistently poses a potential threat to the patient's life, such as congestive heart failure, arrhythmias, sudden death, and thromboembolic events.^[5]^ Therefore, current treatment of DCM is often aimed at controlling symptoms, preventing disease progression and complications.^[6]^ Meanwhile, in China, Traditional Chinese Medicine (TCM) is increasingly used by clinicians as an adjuvant treatment in DCM to reduce the toxic effects of treatment and improve the overall efficacy.^[7]^

Shenmai injection (SMI) is a TCM patent prescription consisting of ophiopogonis radix (*Ophiopogon japonicus* (L. f) Ker-Gawl.) extract and ginseng radix rubra (*Panax ginseng* C. A. Mey.) extract. It has been used to treat dilated cardiomyopathy, pulmonary heart disease, heart failure, angina pectoris, coronary heart disease, and carcinoma.^[8–14]^ Especially, as a complementary and alternative drug, SMI is often used as adjunctive therapy for traditional Western medicine in the treatment of DCM patients. However, there is still a lack of comprehensive and systematic evidence, which urgently requires us to verify its therapeutic efficacy. Therefore, we present a meta-analysis protocol of the therapeutic efficacy of SMI combined with conventional medicine treatment (CMT) versus CMT on DCM.

In this study, we aimed to systematically analyze the published data on clinical efficacy, cardiac function index, and 6-minute walk test of randomized controlled trials (RCTs) investigating SMI combined with CMT in patients with DCM, to compare the auxiliary therapeutic efficacy by meta-analysis.

## 2 Materials and methods

The study protocol has been registered on International prospective register of systematic reviews (PROSPERO), and the study registration ID is CRD42019146369. The protocol followed Preferred Reporting Items for Systematic review and Meta-Analysis Protocols (PRISMA-P) guidelines.^[15]^

### 2.1 Data resources and search strategies

Electronic searches were carried out using PubMed, China National Knowledge Infrastructure (CNKI), Wanfang data, VIP datebase, Embase, the Cochrane Library, and Chinese Biomedical Database (CBM). The databases were searched by 2 investigators independently (from inception to August 8, 2019) and disagreements were settled by discussion with a third reviewer.

We combined the following keywords to identify the publications in several queries: “Shenmai injection OR Shenmai” [Title/Abstract] AND “cardiomyopathy, dilated OR dilated cardiomyopathy OR cardiomyopathy, congestive OR dilated cardiomyopathy 1A OR dilated cardiomyopathy, idiopathic OR idiopathic dilated cardiomyopathy OR congestive cardiomyopathy” [Title/Abstract] AND “randomized controlled trial OR randomized” [Abstract]. Searches were limited to clinical studies published in Chinese and English.

### 2.2 Inclusion and exclusion criteria

The following inclusion criteria were designed to cater to the research theme: the clinical trials involved were RCTs. Patients diagnosed with DCM by the following criteria: Diagnostic Criteria of WHO (World Health Organization)/ISFC (International Society and Federation of Cardiology) cardiomyopathy (Version 1995), or Guidelines for the Diagnosis and Treatment of Dilated Cardiomyopathy in China (Version 1995). Patients in the experimental group received CMT-based therapy with SMI, whereas patients in the control group were treated with CMT-based therapy only. Here, CMT is defined as the administration of oxygen, diuretics, cardiac glycosides, angiotensin-converting enzyme inhibitors or angiotensin II receptor antagonists, nitrates, beta blockers and other western medicines in patients with dilated cardiomyopathy. Such as oral digoxin tablets, furosemide tablets, benazepril hydrochloride tablets, spironolactone tablets, perindopril, metoprolol tartrate tablets, aspirin, isosorbide mononitrate vinegar, enalapril, and so on. The measurement indicators for clinical studies should include the following indicators and the measurement units of the same indicator data are consistent: clinical efficacy (CE), cardiac function index, such as left ventricular ejection fraction (LVEF), left ventricular end-diastolic dimension (LVEDD), left ventricular end-systolic dimension (LVESD), cardiac output (CO), and 6-minute walk test (6MWT).

The following exclusion criteria were intended to remove unreasonable literature and narrow down the literature: if it is an article of one of the following types, it is considered unrelated to the theme: comments, non-clinical experiments, self-control studies, case reports, random method error studies, and reviews. The heart disease caused by other causes, such as coronary heart disease, rheumatic heart disease, viral myocarditis, and congenital heart disease. If the treatment of DCM patients involved other TCM patent prescriptions, or combined with surgery and other medical treatments, it should not be included. If there is a republished clinical literature, only the most recently published publications with large sample sizes and comprehensive studies were retained.

**Figure 1:**
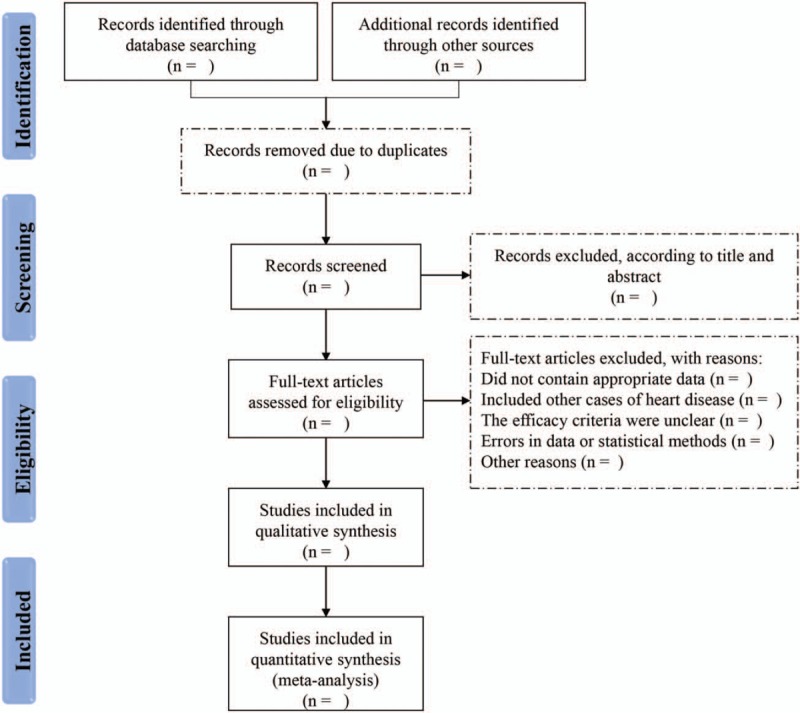
The process of screening for eligible studies.

The detailed screening process is shown in PRISMA flow diagram (Fig. 1).

### 2.3 Quality assessment of included literature

Two investigators independently evaluated the quality and bias risk of methodological in each trial using the method, Assessment of Study Quality, in Cochrane Handbook for Systematic Reviews of Interventions,^[16]^ which consisted of 7 domains (random sequence generation, allocation concealment, blinding of participants and personnel, blinding of outcome assessment, incomplete outcome data, selective reporting, and other bias). If there was a disagreement during the evaluation process, it will be resolved through discussion with a third reviewer.

### 2.4 Data collection and analysis

Two investigators extracted detailed information and available data from the included studies, such as sample size, interventions, duration of intervention, and outcome measures, and organized them in a table for meta-analysis. A third reviewer supervised the process and solved the disagreements through discussion or consensus.

Considering the types of data provided in the original literature and the characteristics of the outcome indicators, we divided these data into dichotomous and continuous variables. For example, clinical efficacy can be considered as dichotomous variables, while levels of the cardiac function index (LVEF, LVEDD, LVESD, CO), and 6MWT as continuous variables. The dichotomous variables were expressed as a risk ratio (RR) with 95% confidence intervals (95% CI), while the continuous variables were expressed as a mean difference (MD) with 95% CI.

Heterogeneity tests were performed by chi-square statistics (Cochrane homogeneity test), while *I*^*2*^ tests are used to quantify the degree of heterogeneity. When *P* ≥ .1 and *I*^2^ ≤ 50%, the data has low heterogeneity and is analyzed using a fixed-effects model (Mantel-Haenszel method); and when *P* < .1 or *I*^*2*^ > 50%, the data has high heterogeneity and is analyzed using a random-effects model (Restricted Maximum Likelihood method). In this case, we sought possible sources of heterogeneity, and attempted to clarify the causes of heterogeneity through subgroup analysis. The potential variables for performing subgroup analysis include: treatment duration, drug dose, and publication date.

Publication bias was explored graphically via funnel plots, and detected via the Egger test and Harbord test.^[17]^

All analyses were performed with Review Manager (RevMan, Version 5.3) and STATA 16.0 software.

## 3 Discussion

It is well known that the premature death caused by cardiomyopathy results in great loss to society, which also become an important public health problem.^[18]^ At present, there is no significant difference between the therapy for DCM and the administration of ordinary heart failure, with the drug base consisting of β-blockers, renin-angiotensin system inhibitors, aldosterone antagonists, and diuretics.^[19]^ Despite optimal medical therapy, the morbidity and mortality are still high, prompting the need for novel treatment options.^[20]^

However, TCM can effectively alleviate the adverse conditions of DCM because of its unique advantages of overall regulation and syndrome differentiation. As an ancient philosophy-based medicine, TCM has become one of the comprehensive treatments for cardiomyopathy.^[21]^ In addition, the combination of integrated traditional Chinese and Western medicine has become a common strategy for clinical treatment of cardiomyopathy in China.^[7]^ SMI has been widely used as adjunctive therapy in clinical practice.^[22]^ Through the implementation of this study protocol, we can comprehensively evaluate the efficacy and safety of SMI in patients with DCM. So as to provide a basis for clinical adjuvant treatment of DCM. Also, the results need to be further confirmed by large sample RCTs with longer follow-ups.

## Author contributions

**Methodology:** Kai Gao.

**Project administration:** Kai Gao.

**Software:** Hao Chen and Lin-Tao Zhao.

**Supervision:** Yan-Ping Song.

**Visualization:** Hai-Wang Zhang and Lin-Tao Zhao.

**Writing – original draft:** Kai Gao, Hao Chen and Hai-Wang Zhang.

**Writing – review & editing:** Anna Song.
